# Feeling Small or Standing Tall? Height Manipulation Affects Speech Anxiety and Arousal in Virtual Reality

**DOI:** 10.1089/cyber.2022.0251

**Published:** 2023-04-14

**Authors:** Anna-Leena Macey, Simo Järvelä, Daniel Fernández Galeote, Juho Hamari

**Affiliations:** Gamification Group, Faculty of Information Technology and Communication Sciences, Tampere University, Tampere, Finland.

**Keywords:** virtual reality, emotion regulation, speech anxiety, emotional response

## Abstract

Social performance situations often constitute one of the most challenging communication tasks across different cultures. In today's work environments, giving presentations and performing in front of others are often essential and expected. Therefore, public speaking anxiety can have a serious impact on an individual's job performance, career choice, and prospects. Contemporary consumer virtual reality hardware has made it possible to practice public speaking anywhere in a safe and private virtual reality environment (VRE). As VREs offer the means to practice real-life scenarios, they also make it possible to go beyond what is “real”; to replace simulations with more dynamic and innovative training environments. Furthermore, with occupational life undergoing a significant shift toward technology-mediated working conditions, innovative tools and methods could also be used during virtually implemented real-time social interactions. This research aimed to study the ways in which an illusion of height, that is, perceived tallness versus perceived shortness, without any visible virtual body or representation, influences state speech anxiety and emotional responses of participants during simulation of a stressful speech task. The experiment followed a strictly controlled between-subject procedure, and both self-reported and psychophysiological data were collected. Results indicate that participants perceiving the illusion of tallness felt less anxious and had lower self-reported arousal compared with participants with the illusion of shortness. This implies that even simple, visual, first-person perspective manipulation of the VRE could help individuals to reduce their stress responses during a task-oriented situation.

## Introduction

Social performance situations, for example, giving a speech in front of an audience, can be highly stressful scenarios, often constituting one of the most challenging communication tasks across different cultures.^1,2^ In today's organizations and work environments, giving presentations and performing in front of others are commonplace and can be essential components of working life. Public speaking anxiety can, therefore, have a serious impact on an individual's job performance, career choice, and prospects.^3,4^

Contemporary consumer VR hardware has a growing number of downloadable applications that make it possible to practice public speaking in safe and private virtual environments. These virtual reality environments (VREs) offer the means to practice real-life scenarios; indeed, VREs have been shown to be effective in replicating real-world behaviors and affective responses.^5,6^ However, these applications concentrate predominantly on skill-based training and rely on an instruction-led approach, yet they also offer the possibility to go beyond what is “real”—to replace simulations with more dynamic and innovative training environments.

Furthermore, occupational life is undergoing a significant shift toward technology-mediated working conditions and interactions, and it is expected that in the future we will be increasingly replacing both physical and video meetings with employment of VR. By manipulating the VRE, users' affective experiences could be enhanced, thus helping them to manage their emotional states and guiding them to feel more empowered and therefore in control of the situation.

The embodied cognition paradigm holds that bodily, affective, cognitive, environmental, and intersubjective states are all heavily intertwined, thus all affecting each other.^7^ Previous studies have demonstrated that adopting open, expansive, and upright postures during task-oriented situations has a positive effect on mood and self-esteem,^8^ feelings of power,^9^ and stress management in general.^8–10^

Studies examining the adoption of similar postures solely before task-oriented situations have also reported increase in felt power in participants.^11,12^ Although some of the results in the earlier works, regarding hormonal and behavioral changes, have not been replicable, the growing body of research does support the finding that an open, expansive, and upright posture held before a task-oriented situation can increase the feelings of personal power.^13^

Open postures project high power, whereas contracted or closed postures project low power.^14,15^ As perceptions of power are associated with expansive nonverbal displays, human height is related to the perceptions of status and dominance.^16^ Similarly, height also produces internal affective manifestations, influencing self-perception and behavior; taller individuals, particularly men, have been found to have higher levels of self-esteem than shorter individuals.^17^

Further research has shown that height influences the outcome of nonverbal confrontations between individuals in social encounters.^16^ In VREs, participants assigned taller avatars were found to behave more confidently and to act more aggressively during a VR negotiation task than participants assigned shorter avatars.^18^

Indeed, avatars in VREs have been found to induce a range of affective responses through situational cues, such as the clothes they are wearing, or other factors connected to appearance.^19^ As vision dominates human perception, visual stimulation plays an important role in generating body illusions.^20^ Previous studies have demonstrated that viewing a virtual body from a first-person perspective (1PP) together with synchronous visuo-tactile stimulation can affect body size perceptions^21,22^ or induce stress.^23^

The primary aim of this research is to study ways in which a simple 1PP illusion of height, that is, perceived tallness versus perceived shortness, without any visible virtual body or representation, influences affective states (state anxiety, emotional response, and physiological arousal) of participants during simulation of a stressful speech task. Using the speech task section of the Trier Social Stress Test^24^ and its VR implementation,^25^ we investigate the possibilities of going beyond a simple simulation by manipulating the VRE and the participant's experience.

Considering the above research, we hypothesize that participants in the tall condition will have less state anxiety during the speech task compared with participants in the short condition (**H1**).

Furthermore, we also expect the height illusion to influence the participants' self-reported emotional responses during the task. More precisely, we expect that the perceived tallness will increase both valence (**H2a**) and dominance (**H2b**) compared with perceived shortness. Finally, we expect that arousal (both self-report and physiological measures) in the tall condition will be reduced compared with the short condition (**H2c**).

## Materials and Methods

### VR equipment and environment

The experiment used the wireless VR headset, Oculus Quest 2 (Meta Platforms Technologies, LLC.), with one handheld controller. Similar to room scale setups, the headset uses positional six degrees of freedom tracking, thus the simulated point of view adjusts to the user's head rotation and position.

**FIG. 1.**
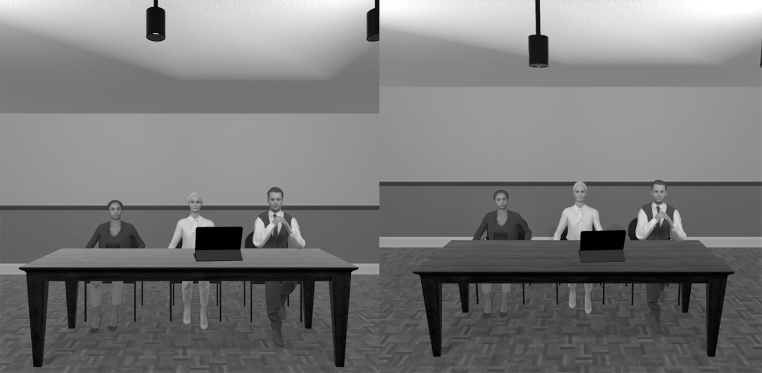
Screenshots of participant views (short and tall) in the virtual reality environment.

The VRE used for this experiment was created using the Unity game engine (Unity Technologies) and consisted of three rooms: (a) a plain empty room for baseline measurement; (b) a waiting room for the speech preparation phase; and (c) a room for the speech task with a virtual three-member evaluation committee consisting of agents representing a variety of ethnicities, genders, and age groups; throughout the speech, their facial expressions remained neutral (Fig. 1).

The agent sitting in the middle gave prerecorded and time-activated instructions to the participants, for example, encouraging them to continue if they remained silent for a period of 5 (±) seconds. Agents did not have any facial movements, only small cyclical movements such as breathing and subtle head and foot movements, similar to the study by Wallergård et al.^25^ During the speech, the camera position of the head-mounted display (HMD) was either raised or lowered 15% from a default height, thereby creating a 1PP illusion of the participant being either short or tall.

Throughout the simulation, the participants did not have any visible body. The users' default height was set at the average eye-level height of the agents when in a standing position.

### Psychophysiological measurement

The wearable wristband, Empatica E4 (Empatica Srl, Milan, Italy), hereafter E4, was used during the experiment to measure the heart rate (HR) and electrodermal activity (EDA) in participants. E4 has been designed for research purposes and contains a photoplethysmography (PPG) sensor to provide the blood volume pulse from which HR data are derived.

For measuring EDA, E4 uses two dry electrodes positioned on the wrist. As participants held the controller in their right hand, the wristband was worn on their left wrist to minimize possible movement artifacts caused by the use of the controller.

### Participants

Participants were recruited through a faculty mailing list and by advertising throughout the university campuses. The sample (*n* = 61) consisted of 26 females, 34 males, and one other, with ages ranging from 20 to 55 years; it was a multicultural generally healthy group of mainly university students and employees, of which 10 were native English speakers (Table 1).

**Table 1.** Demographic Information

Age, years		
Range	20–55	
Mean	32	
*SD*	8.056	
	n	*%*
Gender		
Female	26	42.6
Male	34	55.7
Other	1	1.6
Nationality		
(The) United States	4	6.6
Australia	1	1.6
Austria	1	1.6
Bangladesh	4	6.6
Brazil	1	1.6
China	4	6.6
Croatia	1	1.6
Finland	16	26.2
Germany	4	6.6
Greece	1	1.6
India	3	4.9
Iran	2	3.3
Ireland	1	1.6
Jordan	2	3.3
Lithuania	1	1.6
Mexico	1	1.6
Nicaragua	1	1.6
Norway	1	1.6
Pakistan	2	3.3
Peru	1	1.6
Russia	5	8.2
South Korea	2	3.3
Syria	1	1.6
Vietnam	1	1.6

*SD*, standard deviation.

This research conforms to the standard ethical guidelines and responsible conduct of research set by the Finnish National Board on Research Integrity (TENK). The research was completed in accordance with the Declaration of Helsinki, as revised in 2013, and has passed the ethics committee review of Tampere University.

### Procedure

During registration, participants provided demographic information, received information about the nature of the research and data privacy, and were randomly assigned to one of the two conditions, short or tall. When arriving at the laboratory, participants received a comprehensive briefing about the procedure, including their right to withdraw, before providing written consent.

To obtain unbiased data, participants were informed that the nature of the research simply concerned the use of VR as a public speaking platform, and the height manipulation was not disclosed before participation. Finally, the E4 wristband measuring HR and EDA and the HMD were fitted.

After entering the VRE, baseline measures of HR and EDA were recorded in room (a) for a period of 8 minutes, after which participants completed the Self-Assessment Manikin (SAM) questionnaire. In room (b), they received information and guidance for the speech task ahead. Their task was to adopt the role of a job applicant and prepare a 5-minute speech describing why they would be perfect candidates for their dream job. After 5 minutes of preparation, participants were transferred into room (c), where they delivered their speech to an evaluation committee.

After the speech, participants were directed back to room (a) to complete the post-task SAM scale and the Public Speaking Anxiety Scale (PSAS). Participants were then fully debriefed and received a cinema ticket as compensation for their participation.

### Measures

Two self-report measures and physiological measures were used to make between-condition comparisons on emotional states during the speech task. Both self-reports were in English as the experiment included multicultural participants.

PSAS^26^ is a 17-item instrument measuring cognition, behaviors, and physiological manifestations of speech anxiety. In this study, the statements of the scale were amended to specifically measure the levels of participants' state anxiety experienced *during* the speech task; for example, “Giving a speech is terrifying” was amended to “Giving the speech was terrifying.”^27^ Statements were rated on a 5-point Likert scale (from 1: not at all, to 5: extremely).

SAM^28^ is a three-item instrument measuring emotional responses to stimuli using visual representations of valence, arousal, and dominance. Each of these dimensions is portrayed by nine drawn characters depicting a range of responses for each scale.

To support self-reported assessments of emotional states, psychophysiological measures were also utilized.^29^ To assess the physiological arousal of participants during the experiment, HR^30^ and EDA^31,32^ were measured.

### Data processing and analysis

HR physiological data from Empatica E4 were processed with the Kubios HRV Standard^33^; unfortunately, due to movement artifacts caused by many participants' lively hand gestures while speaking, R-peaks could not be detected reliably enough from the data for majority of participants and consequently the data were not of sufficient quality to report HR results.

EDA data were processed using Ledalab 3.4.9 and MATLAB R2022a.^34^ For 19 participants, EDA data quality was not sufficient and had to be omitted from the final analysis, leaving sample sizes of 23 for short and 19 for tall condition groups. After segmentation and artifact removal, a continuous decomposition analysis^35,36^ was performed to separate tonic and phasic components.

The rate and mean amplitude of nonspecific skin conductance responses (NSSCRs) over a 5-minute test period were used in the analysis; a 5-minute period (3–7 minutes) from the baseline measurement was used as a reference. NSSCRs were used instead of the mean skin conductance level, associated with both tonic task performance-related stress and arousal, during the period as they can be considered less sensitive to challenges posed by mediocre data quality.

For self-report measures, in five cases, individual items were removed from the data for participants who indicated that they had trouble understanding the meaning of the self-report item, but no outlier removal was performed. For descriptive statistics, see Table 2.

**Table 2.** Group Descriptives

Variable	Condition	*n*	Mean	Median	SD	SE
Valence	Short	31	6.1	6	1.8	0.322
	Tall	30	6.8	7	1.71	0.312
Arousal	Short	29	4.93	5	1.889	0.351
	Tall	29	3.97	4	1.658	0.3079
Dominance	Short	31	5.39	6	1.63	0.292
	Tall	30	6.1	6	1.79	0.326
Public Speaking Anxiety: Cognitive	Short	31	2.58	2.5	0.866	0.156
	Tall	30	2.1	2	0.635	0.116
Public Speaking Anxiety: Behavioral	Short	31	2.4	2.33	0.854	0.153
	Tall	30	1.82	1.67	0.488	0.0891
Public Speaking Anxiety: Physiological	Short	31	2.35	2.4	0.738	0.132
	Tall	30	2.04	2	0.471	0.086
NSSCR rate	Short	23	108.2	110	39.5	8.23
	Tall	19	94	90	34.2	7.84
NSSCR amplitude	Short	23	0.292	0.230	0.165	0.03
	Tall	19	0.289	0.236	0.133	0.03

NSSCRs, nonspecific skin conductance responses; *SE*, standard error of the median.

Statistical analyses were conducted using Jamovi 2.2.2.0^37^ and figures plotted with R 4.2.^38^ First, scale reliabilities were assessed for PSAS subscales, and after removing one low-loading item from the behavior subscale that was already identified as problematic based on participant feedback, all Cronbach's alphas were found to be above 0.7 (cognitive α = 0.882, behavioral α = 0.782, and physiological α = 0.706). Second, the two condition groups were compared to confirm that they were not significantly different in self-reported participant height (*t* = 0.551, *df* = 57, *p* = 0.583) or in public speaking experience (*U* = 399, *p* = 0.309).

The Mann-Whitney *U* test (MWU) was used for the main analysis as Shapiro–Wilk tests revealed that some parts of the data violated assumptions of normality. Homogeneity of variance between groups was tested using Levene's test and found to be equal (*p* > 0.05). For orthogonal tests, the MWU was first conducted to compare baseline measurements to confirm that the test groups were sufficiently similar (all *p* > 0.05).

Next, the Wilcoxon W test was performed to check if the baseline recording and test period were different within subject for SAM ratings (*p* < 0.001 for arousal and *p* > 0.05 for valence and dominance) and EDA (both rate and amplitude of NSSCRs *p* < 0.001), that is, the stress task was stressful compared with the baseline regardless of height manipulation (Table 3).

**Table 3.** Wilcoxon W Test with Pretest and Post-Test Scores

Measure	Statistic	*p*	Effect size (*r_rb_*)
Valence	613.5	0.085	0.297
Arousal	187.0	<0.001	−0.622
Dominance	470.5	0.772	−0.049
NSSCR rate	0.0	<0.001	−1.000
NSSCR amplitude	23.0	<0.001	−0.944

*r_rb_*, rank biserial correlation.

Finally, different variables were plotted comparing the experimental groups before a series of one-sided MWUs, in line with the preregistered hypotheses, were conducted (Table 4).

**Table 4.** Mann-Whitney *U* Test

Variable	Value	*p*	Effect size (*r_rb_*)
Valence^a^	362	0.067	0.222
Arousal^b^	291	0.021	0.308
Dominance^a^	364	0.07	0.217
Public Speaking Anxiety: Cognitive^b^	322	0.019	0.309
Public Speaking Anxiety: Behavioral^b^	273	0.003	0.414
Public Speaking Anxiety: Physiological^b^	359	0.062	0.229
NSSCR^b^ rate	154	0.053	0.295
NSSCR^b^ amplitude	210	0.589	0.039

^a^ H_a_ μ_Short_ < μ_Tall_.

^b^ H_a_ μ_Short_ > μ_Tall_.

## Results

**FIG. 2.**
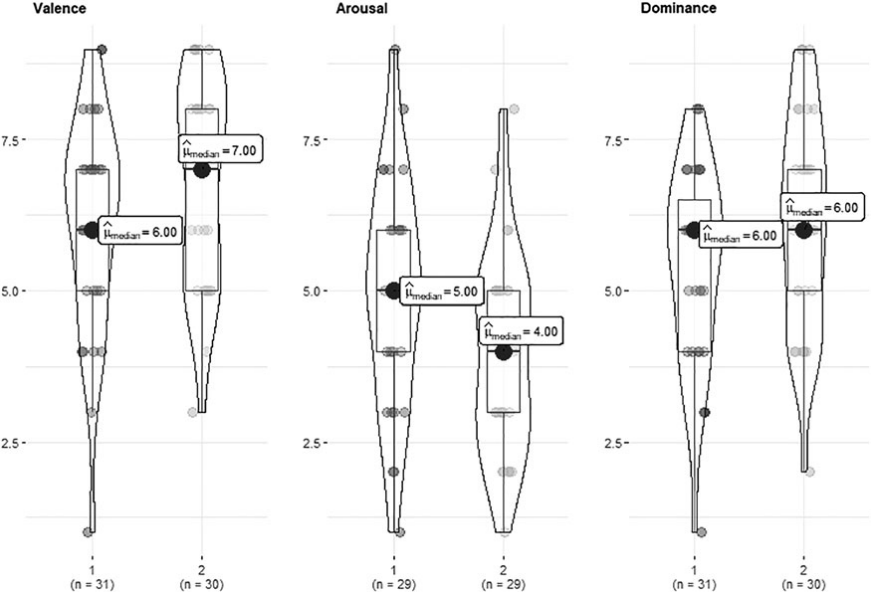
Self-Assessment Manikin. *Note:* 1, Short; 2, Tall.

**FIG. 3.**
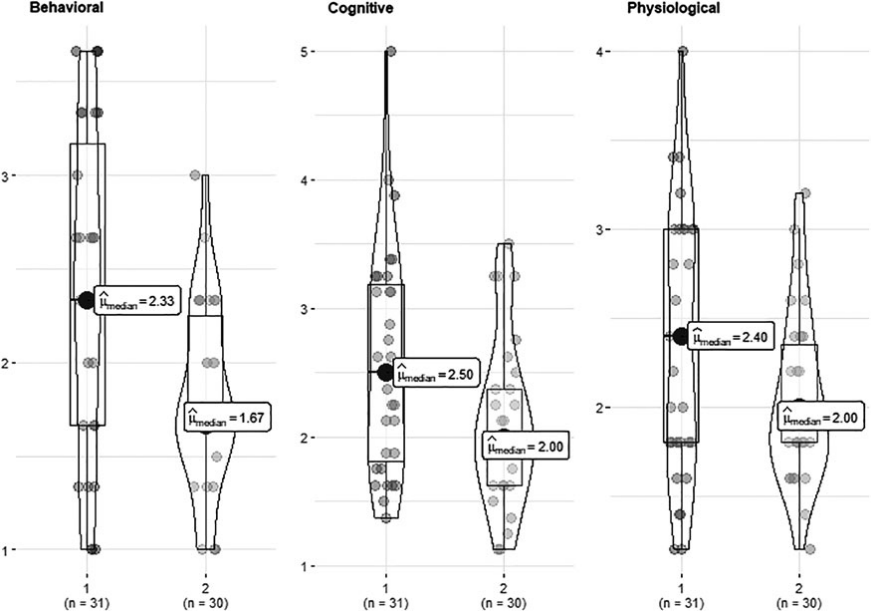
Public Speaking Anxiety Scale. *Note:* 1, Short; 2, Tall.

**FIG. 4.**
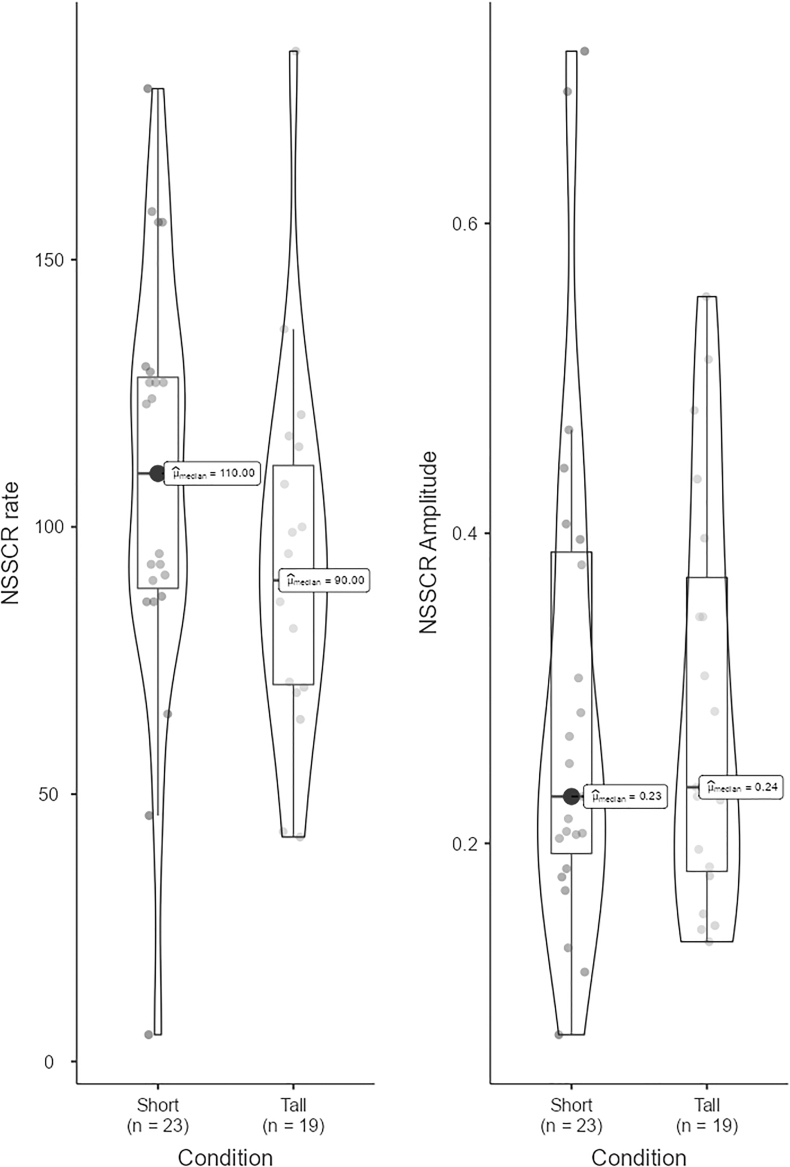
Nonspecific skin conductance responses.

Participants in the tall condition reported lower arousal (*U* = 291, *p* = 0.021, *r_rb_* = 0.308) (Fig. 2 and Table 4) and public speaking anxiety (cognitive [*U* = 322, *p* = 0.019, *r_rb_* = 0.309]; behavioral [*U* = 273, *p* = 0.003, *r_rb_* = 0.414]; and physiological [*U* = 359, *p* = 0.062, *r_rb_* = 0.229]) (Fig. 3 and Table 4) with moderate effect sizes than those in the short condition. Physiological arousal, as assessed with the more robust NSSCR rate (NSSCRr), was similarly lower in the tall condition compared with short condition as self-reported arousal (*U* = 154, *p* = 0.053, *r_rb_* = 0.295) (Fig. 4 and Table 4). No difference was found in the NSSCR amplitude (NSSCRa; *U* = 210, *p* = 0.589, *r_rb_* = 0.04), which can be more sensitive to issues with data quality.

In short, the results support our main hypotheses that participants in the tall condition felt less state speech anxiety (cognitive and behavioral components) (H1) and lower arousal (H2c) compared with those in the short condition. Interestingly, while differences in self-reported arousal were significant (*p* = 0.021), the physiological component of state speech anxiety (SSA-P; *p* = 0.062), NSSCRr (*p* = 0.053), and NSSCRa (*p* = 0.589) were not statistically significant. Nevertheless, both SSA-P and NSSCRr approached the standard threshold (*p* < 0.05).

Regarding valence and dominance, no statistically significant differences were observed between baseline and test conditions, nor between short and tall conditions during the task (Fig. 2 and Table 4): valence (*U* = 362, *p* = 0.067, *r_rb_* = 0.222) and dominance (*U* = 364, *p* = 0.070, *r_rb_* = 0.217). Therefore, H2a and H2b are not supported.

## Discussion

The results of this study add to the growing body of literature supporting the use of bodily expansion to positively influence stress responses during a task-oriented situation.^8–10^ In this work, we particularly tested whether this effect can be replicated through novel VR and 3D technologies; we found that modifying the users' perceived height affected participants' self-reported arousal and the cognitive and behavioral components of state speech anxiety.

These results support previous findings of VRE's effectiveness in inducing a range of affective responses. They also indicate the importance of visual stimulation in generating body illusions in VR. Previous studies have generated embodiment illusions^21,22^ and generated stress in participants^23^ by using multisensory modalities, i.e. through 1PP view of virtual bodies alongside synchronous visuo-tactile stimulation.

The results of this study indicate that just a simple, visual 1PP cue can be enough to generate an illusion of height in VR, even without any visible virtual representations. Furthermore, this study implies that only a simple visual illusion is sufficient to influence anxiety and arousal during a stressful task.

Interestingly, the findings of this study are also in line with earlier research on expansive nonverbal displays in the physical world and the ability of these displays not just to produce internal affective manifestations but also to help with stress responses when held during a task-oriented situation.^8–10^ Our results indicate that even an experience of virtual expansion, an illusion of being taller, can reduce stress-related anxiety and arousal. However, our findings of reduced arousal were not completely consistent, indicating the need for further investigation.

Differences in self-reported arousal were statistically significant, while the other measures, the SSA-P and EDA (NSSCRr/NSSCRa), did not reach this threshold. SSA-P and NSSCRr, however, approached the standard threshold of statistical significance. This inconsistency demonstrates the importance of using diverse measures where possible to balance subjective and objective data; relying solely on either self-reports or physiological data may run the risk of not capturing the whole picture.

Our expectation was that the height illusion would lead to higher valence and feelings of dominance during a stress-inducing speech task. This would have been in line with previous findings^8,16^; however, results for valence and dominance were not statistically significant. This may be explained by underlying issues with the SAM scale; the dominance dimension, in particular, has been highlighted as not showing consistent effects across studies^28^; alternatively, this inconsistency could also be due to the strong correlation between the dominance and valence dimensions.^39,40^ Consequently, the relationship between valence and dominance and a virtual height illusion will require further investigation.

The results of this study indicate that even simple manipulation of the VRE could help individuals to reduce their arousal and anxiety during a task-oriented situation. These findings have the potential to be applied to a range of affective VR training simulations, which address both work-based social interactions and performance scenarios. As it is expected that in the future we will increasingly be replacing both physical and video meetings by employing VR, the findings have the potential to offer new innovative tools and methods to manage stress during real time, virtually implemented work-based tasks, or social interactions; for example, virtual pitching sessions or job interviews.

These benefits are not limited only to organizational contexts. The COVID-19 pandemic and ongoing climate crisis have increased the popularity of VR as a social medium,^41,42^ therefore these findings have the potential to be applied to a range of VREs used for all forms of social interactions. As such, these results highlight the dynamic and innovative affordances of VR technology as a means to provide users with easily adjustable tools that can privately support their stress management.

This study captured the effect on participants who were not fully aware of the height manipulation and the explicit aims of the research. Further investigation is needed to ascertain if the effects of this manipulation are also applicable to participants fully conscious of both the manipulation and the aim of the tall condition to increase empowerment during a stressful situation.

There are limitations to this study, notably that the moderate sample size and the use of convenience sampling mean that results may not be generalizable. The total number of participants was 61; however, many VR studies use similar or often even smaller sample sizes and have yielded meaningful results. Additionally, the sense of presence was not measured during the VR exposure, meaning that the degree to which participants felt immersed in the VRE was not included in the analysis.

While perceived immersion has the potential to add more detailed understanding of the mechanisms at work, even without this measure, clear effects were observed. As such, it is recommended that future work incorporates some measure of presence as a form of control variable, thereby adding more nuance to results.

One of the strengths of this current study was that the experiment was well controlled and focused on a single independent variable. This can be considered a merit of this study as experiments conducted in VR research are often either more complex or more naturalistic. Furthermore, while this study used convenience sampling, it is worthy of note that the sample used in this study consisted of individuals of a wide range of nationalities, representing almost every continent. Although it does not make the results any more generalizable, it gives an indication that the effects observed in this study are similar despite the cultural background.

Finally, due to challenges experienced with the physiological measuring device, the HR data were not of sufficient quality and could not be used; similarly, although the EDA data were generally of much higher quality, 19 participants were excluded from the analysis as the device did not capture data to the required level. However, these are existing challenges with wearable devices.

Increasingly popular PPG sensors, used also in E4 wristbands as an HR monitoring technique, are very sensitive to motion artifacts, for example, hand movements.^43,44^ This is also the case with electrodes measuring EDA as they require permanent skin contact to provide undisturbed data, while sensors positioned on the fingers or palms offer greater conductance. As such, the fact that the E4 uses both dry electrodes and is positioned on the wrist could potentially reduce the quality of the gathered data.^44,45^

## Conclusions

The results of this study indicate that simple manipulation of the VRE, a height illusion created only by raising or lowering the HMD camera angle, without any visible virtual representations, affects the participants' self-reported state speech anxiety and arousal. These findings also indicate the importance of visual stimulation, particularly when generating body illusions in VR. Our results demonstrate that an experience of virtual expansion, that is, an illusion of being taller, can have similar effects as those observed during research in purely physical domains.

These findings have the potential to be applied to a range of affective VR training simulations and performance scenarios as well as to offer new innovative tools and methods to manage stress during real-time, virtually implemented work-based tasks or social interactions.

## Data Availability Statement

The data supporting the conclusions of this article will be made available by the authors, without undue reservation. The procedure and hypotheses have been preregistered at the Open Science Framework (OSF): DOI: 10.17605/OSF.IO/HAZ5T.
